# Reliability of quadriceps surface electromyography measurements is improved by two vs. single site recordings

**DOI:** 10.1007/s00421-017-3595-z

**Published:** 2017-04-08

**Authors:** T. G. Balshaw, A. Fry, T. M. Maden-Wilkinson, P. W. Kong, J. P. Folland

**Affiliations:** 1 School of Sport, Exercise, and Health Sciences, Loughborough University, Leicestershire, LE11 3TU UK; 2 Institute for Sports Research, Nanyang Technological University, Singapore, Singapore; 3 Arthritis Research UK Centre for Sport, Exercise and Osteoarthritis, Loughborough University, Leicestershire, UK; 4 Faculty of Health and Wellbeing, Collegiate Campus, Sheffield Hallam University, Sheffield, UK; 5 Physical Education and Sports Science Academic Group, National Institute of Education, Nanyang Technological University, Singapore, Singapore

**Keywords:** Voluntary muscle contraction, Peripheral nerve stimulation, Knee extension, Coefficient of variation, Intraclass correlation coefficient, Evoked muscle response

## Abstract

**Purpose:**

The reliability of surface electromyography (sEMG) is typically modest even with rigorous methods, and therefore further improvements in sEMG reliability are desirable. This study compared the between-session reliability (both within participant absolute reliability and between-participant relative reliability) of sEMG amplitude from single vs. average of two distinct recording sites, for individual muscle (IM) and whole quadriceps (WQ) measures during voluntary and evoked contractions.

**Methods:**

Healthy males (*n* = 20) performed unilateral isometric knee extension contractions: voluntary maximum and submaximum (60%), as well as evoked twitch contractions on two separate days. sEMG was recorded from two distinct sites on each superficial quadriceps muscle.

**Results:**

Averaging two recording sites vs. using single site measures improved reliability for IM and WQ measurements during voluntary (16–26% reduction in within-participant coefficient of variation, CV_W_) and evoked contractions (40–56% reduction in CV_W_).

**Conclusions:**

For sEMG measurements from large muscles, averaging the recording of two distinct sites is recommended as it improves within-participant reliability. This improved sensitivity has application to clinical and research measurement of sEMG amplitude.

## Introduction

Surface electromyography (sEMG) is used extensively to measure the electrical activity within skeletal muscles in clinical and research applications, including: the investigation of neurological diseases (Martin et al. 2006; Perrin et al. 2011; Rissanen et al. 2007); the assessment of motor control and muscle dysfunction (Birch et al. 2000; Nederhand et al. 2002; MacDonald et al. 2009); and the evaluation of rehabilitation/exercise interventions (Aagaard et al. 2002; Buckthorpe et al. 2015; Fimland et al. 2010). Despite the relative ease with which sEMG measurements can be performed, there are numerous technical and methodological issues that are recommended to maximise signal fidelity and measurement reliability (De Luca 1997). These considerations include skin preparation (Cram and Rommen 1989), sensor placement (Hermens et al. 2000; Rainoldi et al. 2004) and the use and selection of normalisation methods (Burden 2010; Balshaw and Hunter 2012; Buckthorpe et al. 2012). Nonetheless, despite careful attention to these issues the reliability of absolute sEMG amplitude recording during both voluntary and evoked (involuntary) contractions remains modest (Ball and Scurr 2010; Buckthorpe et al. 2012; Rota et al. 2013). Therefore, methods to further improve the reliability of sEMG measurements of neuromuscular activity are desirable.

The between-session reliability of sEMG measurements are sensitive to any variations in volume conduction (Rutkove 2007), skin impedance (Hermens et al. 2000), and the skin-electrode interface at that particular recording site (Huigen et al. 2002), even if sensor location is precisely replicated. Recordings from a single site may be particularly susceptible to these sources of noise. Furthermore, recordings from a single sEMG sensor measure electrical activity from a relatively minor fraction of large muscles (e.g. the constituent members of the quadriceps femoris muscle group). In contrast, recording sEMG from more than one sensor/site, when averaged, may provide a more robust and reliable measurement of neuromuscular activity that is less susceptible to the noise present at a single site and provide a better representation of electrical activity within the whole muscle (Rash and Quesada 2006). Therefore, we hypothesised that two sensors placed at distinct locations on the same muscle, to derive an average, may improve the between-day reliability of sEMG measurements and facilitate a more stable measure of neuromuscular activity across a large muscle. Measuring a larger proportion of the motor unit pool and the statistical effect of increasing the number of measurements performed might be expected to increase reliability. If this were the case the use of two site sEMG recording might offer greater reliability, and thus also sensitivity, of sEMG measurements for clinical and research applications. Indeed, recent studies have adopted the approach of averaging sEMG from two distinct sensor locations on the same muscle (Fry and Folland 2014; Haider and Folland 2014; Balshaw et al. 2016). However, the effect of using the average of two distinct sEMG recording sites, opposed to one, on the reliability of sEMG amplitude measurements has not been investigated.

The purpose of this study was to compare the between-session reliability of sEMG amplitude measurements from single vs. mean of two sEMG recording sites. The primary measure of reliability was within-participant absolute reliability (coefficient of variation, CV_W_) and the secondary measure was between-participant relative reliability (intraclass correlation coefficient, ICC). Comparisons were made for each of the individual superficial quadriceps muscles as well as for the quadriceps as a whole (averaged based on either 1 or 2 electrode recording sites per individual muscle) during voluntary (maximum and submaximum) contractions and electrically evoked maximal M-waves (M_MAX_).

## Materials and methods

### Participants

Twenty healthy males (mean ± SD, age 22 ± 4 years, height 1.80 ± 0.06 m, body mass 75 ± 9 kg) who were not involved in any systematic physical training provided written informed consent prior to participation in this study, which was approved by Loughborough University Ethical Advisory Committee. Participants had low to moderate physical activity levels [2106 ± 2248 METmin wk^−1^; international physical activity questionnaire (IPAQ): short format (Craig et al. 2003)] and no history of systematic strength and/or power training.

### Overview

**Fig. 1:**
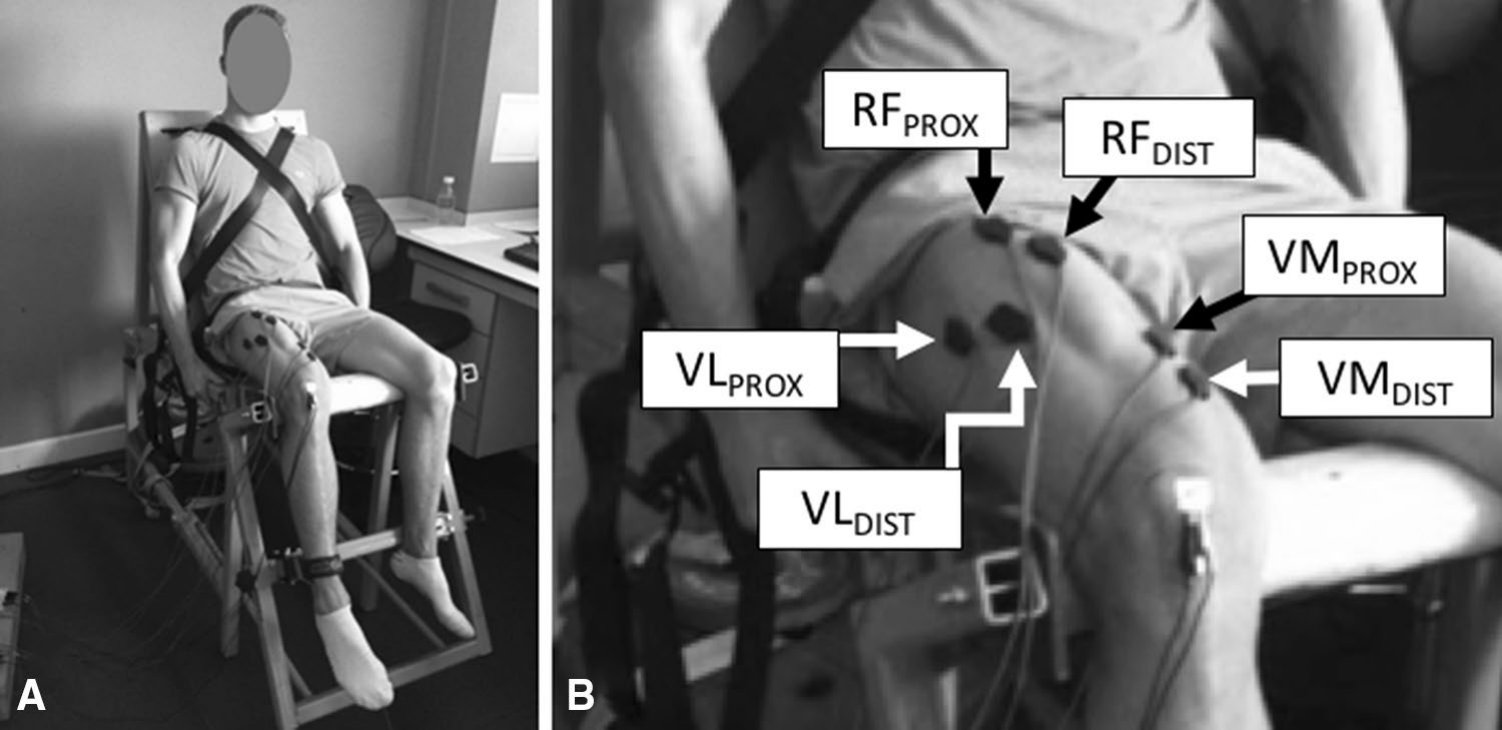
**a** The custom-built rigid isometric testing chair that was used to perform voluntary and evoked contractions; and **b** the six quadriceps surface EMG (sEMG) recording sites (two on each superficial quadriceps muscle) used to derive single and two site measures for individual muscles (*VM* vastus medialis, *VL* vastus lateralis, *RF* rectus femoris) and the whole quadriceps. The two sEMG recording sites over each individual muscle were offset both proximally (_PROX_)/distally (_DIST_) and medio-laterally

Participants attended three test sessions (one familiarisation and two identical test sessions), each at a consistent time of day (12:00–18:00) and separated by 7 days. Participants were instructed to abstain from caffeine, alcohol, and strenuous exercise for 36 h prior to each visit. Throughout each session, participants were seated on a rigid custom-made isometric knee extension dynamometer (Fig. 1a) with knee and hip joint angles of 120**°** and 100**°** (180**°** representing full extension), respectively. Knee extension force and quadriceps sEMG were recorded throughout the two test sessions, whilst participants performed knee extensor contractions of the dominant leg: submaximum and maximum voluntary contractions (MVCs), and electrically evoked maximal twitch contractions with M_MAX_ responses. During the familiarisation session participants completed the same contractions but no data were recorded.

### Knee extension force

The configuration of the isometric dynamometer was determined during the familiarisation session and replicated for subsequent test sessions. Adjustable waist and shoulder straps were used to ensure participants were secured firmly in the chair, and prevented extraneous movements. An ankle strap (35 mm width reinforced canvas webbing) was placed proximal to the ankle (15% of tibial length above the medial malleolus), positioned perpendicular to the tibia and in series with a calibrated S-beam strain gauge (Force Logic, Swallowfield, UK). The analogue force signal from the strain gauge was amplified (×370) and sampled at 2000 Hz using an external A/D converter (Micro 1401, CED Ltd., Cambridge, UK) interfaced with Spike 2 computer software (CED Ltd., Cambridge, UK). In offline analysis, force data were low-pass filtered at 500 Hz using a fourth-order zero-lag Butterworth filter. As a custom built dynamometer with a low noise strain gauge (noise range: <0.1 Nm) was used in this study rather than a commercial dynamometer (noise range ~5 Nm) minimal filtering was applied to the force signal (Maffiuletti et al. 2016). Force data were gravity corrected by subtracting baseline force from active force measures.

### Electromyography

sEMG was recorded using two Delsys Bagnoli-4 acquisition systems (Delsys Inc., Boston, MA). Two separate double differential wired sEMG sensors (Bagnoli DE-3.1, Delsys, Boston, MA) were placed over the belly of each superficial quadriceps muscle at specific percentages of thigh length (greater trochanter to lateral knee joint space) from the superior border of the patella as follows: vastus medialis (VM), 35% (VM_PROX_) and 25% (VM_DIST_); vastus lateralis (VL), 55% (VL_PROX_) and 45% (VL_DIST_); and rectus femoris (RF), 65% (RF_PROX_) and 55% (RF_DIST_) (Fig. 1b). The proximal and distal sensors on each muscle were offset medio-laterally from the longitudinal mid-line of the muscle belly by 10 mm, and aligned parallel to the presumed orientation of the muscle fibres. The separation of the two electrodes placed over each muscle (proximal–distal and medio-lateral) was used to avoid/minimise recording from the same muscle fibres/motor units. The proportions of thigh length chosen to position sensors at were selected to avoid the innervation zones on each muscle (Rainoldi et al. 2004). A reference electrode was situated over the patella of the same leg. The sEMG signals were amplified (×1000; double differential amplifier, EMG signal bandwidth: 20–450 Hz), sampled at 2000 Hz and synchronised with the force data using the same data acquisition equipment.

### Protocol

Following a series of submaximum unilateral isometric knee extension warm-up contractions [50% (×3), 75% (×2), and 90% (×1) of perceived maximum effort] experimental measurements were completed in the following order.

### Maximum voluntary contractions

Participants performed four MVCs and were instructed to “push as hard as possible” for 3–5 s during MVCs and rest for ≥30 s between each effort. A force–time curve with a horizontal cursor indicating the greatest force obtained within the session was displayed for biofeedback, and verbal encouragement was provided during all MVCs. Knee extension maximum voluntary force (MVF) during each measurement session was taken from the individual MVC that produced the greatest instantaneous force and was calculated as the mean force over a 500 ms window (250 ms either side of the greatest instantaneous force). Root mean square (RMS) EMG for the same 500 ms epoch at MVF (EMG_MVF_) was calculated for each quadriceps sEMG sensor before determining single and two site measurements (see Data analysis and statistics).

### Submaximum voluntary contractions

Once MVF had been established a horizontal cursor indicating a target force level of 60% MVF was placed on the force–time curve to ensure the desired force level was achieved during a single submaximum contraction where participants were required to match and hold this target force level for ~5 s. Mean force was calculated for a 500 ms time period when there was a steady plateau in force at ~60% MVF. RMS EMG was measured for this same epoch (EMG_60%MVF_) for each quadriceps sEMG sensor before calculating single and two site measurements (see Data analysis and statistics section).

### Evoked twitch contractions with M_MAX_ responses

Femoral nerve stimulation was conducted with a constant current variable voltage stimulator (DS7AH; Digitimer Ltd., Welwyn Garden City, UK), cathode probe (1 cm diameter, Electro-Medical Supplies Ltd., Wantage, UK), and anode electrode (7 × 10 cm carbon rubber electrode; Electro-Medical Supplies Ltd., Wantage, UK). The cathode and anode were coated with electrode gel and securely taped to the skin over the femoral nerve in the femoral triangle and over the greater trochanter, respectively. Cathode location was determined by delivering single electrical impulses (square wave-pulses of 0.2 ms duration, ≥12 s apart) to identify the position that elicited the greatest submaximum twitch response. Thereafter, current intensity was increased until plateaus in peak twitch force and M-wave peak-to-peak (P–P) amplitude were observed. The current intensity was then increased to a supra-maximal level (+50%) and a further three single impulses (15 s apart) were delivered to elicit three M_MAX_ responses. M_MAX_ P–P amplitude and M_MAX_ area were averaged across the three supra-maximal twitch contractions for each of the individual sEMG recording sites. M_MAX_ area was calculated as the cumulative area from EMG onset (after stimulation artefact) to the point where the signal returned to baseline. Peak force from the three supra-maximal twitches was also averaged (twitch peak force).

### Data analysis and statistics

All sEMG measurements during the voluntary (EMG_MVF_, EMG_60%MVF_) and evoked (M_MAX_ area, M_MAX_ P–P) contractions were first determined for each individual test session. Measurements from each of the six recording sites, two on each of the VL, VM and RF, were considered individually as single site measurements. Two site measurements for each individual muscles were averaged across the two individual sites (e.g. VM_TWO_ = [VM_PROX_ + VM_DIST_] / 2). To calculate whole quadriceps (WQ) values using only single site recordings from each individual muscle, averages were determined from the three proximal and the three distal recording sites of the individual muscles (e.g. WQ_SINGLE−PROX_ = [VM_PROX_ + VL_PROX_ + RF_PROX_]/3). Whole quadriceps sEMG measurements based on two recording sites per muscle were averaged across the two site measurements from each individual muscle (e.g. WQ_TWO_ = [VM_TWO_ + VL_TWO_ + RF_TWO_]/3). Data are reported as mean ± SD. SPSS Version 22.0 (IBM Corp., Armonk, NY) was used to conduct all statistical analysis and statistical significance was set at *P* ≤ 0.05.

The primary outcome measure was within-participant coefficient of variation (CV_W_, [SD/mean] × 100) of sEMG amplitude values from each test session as a measure of absolute reliability. CV_W_ values were calculated for single and two site measurements from each individual muscle and were averaged to calculate representative single site [CV_W_ of IM_SINGLE_ mean = (CV_W_ of VM_DIST_ + CV_W_ of VM_PROX_ + CV_W_ of VL_DIST_ + CV_W_ of VL_PROX_ + CV_W_ of RF_DIST_ + CV_W_ of RF_PROX_)/6] and two site measurements that were not site/muscle specific [CV_W_ of IM_TWO_ mean = (CV_W_ of VM_TWO_ + CV_W_ of VL_TWO_ + CV_W_ of RF_TWO_)/3]. Similarly, single site whole quadriceps CVw values were averaged to provide a representative CV_W_ value [CV_W_ of WQ_SINGLE_ mean= (CV_W_ of WQ_SINGLE−DIST_ + CV_W_ of WQ_SINGLE−PROX_)/2] independent of proximal/distal sites. CV_W_ values were interpreted as “acceptable” <12%, “intermediate” 12–20%, or “unacceptable” >20% (Albertus-Kajee et al. 2011). Shapiro–Wilk tests were used to assess the normality of the sEMG data and CV_W_ values for each single site and two site variable. Several of the variables were not normally distributed and consequently non-parametric statistical tests were used. Wilcoxon signed-rank tests were conducted to compare CV_W_ values between: single and two site measurements; and individual muscle vs. whole quadriceps measurements.

The secondary outcome measure was the ICC. As several of the sEMG variables were not normally distributed they were log-transformed to meet the assumptions of the parametric ICC prior to this statistical test being conducted. ICC values were interpreted as ‘‘very high’’ 0.9–1.0, ‘‘high’’ 0.7–0.9, “moderate” 0.5–0.7, “low” 0.3–0.5, “negligible” 0.0–0.3 (Hinkle et al. 2002). The interpretation of ICC values was done broadly by comparing mean ICC values across several variables (e.g. ICC of IM_SINGLE_ mean vs. ICC of IM_TWO_ mean) and when these were consistently higher (e.g. across most of the voluntary and evoked measures), were considered qualitatively different.

## Results

### Reliability of voluntary and evoked force

MVF displayed CV_W_ values of 2.6 ± 2.0% (acceptable) and an ICC value of 0.977 (very high) with no difference between test days (session 1: 807 ± 126 N, session 2: 818 ± 129 N; Wilcoxon *P* = 0.159). Force production during submaximum contractions displayed CV_W_ values of 2.3 ± 1.5% (acceptable) and an ICC value of 0.984 (very high) with no difference between tests days (session 1: 478 ± 75 N, session 2: 482 ± 74 N; Wilcoxon *P* = 0.247). The submaximum contractions were a consistent proportion of MVF on each test day (session 1: 59.3 ± 2.3%MVF, session 2: 59.0 ± 2.4%MVF). Twitch peak force displayed CV_W_ values of 6.3 ± 5.0% (acceptable) and an ICC value of 0.955 (very high) with no difference between test days (session 1: 144 ± 35 N, session 2: 152 ± 41 N; Wilcoxon *P* = 0.052).

### Reliability of sEMG measurements

**Fig. 2:**
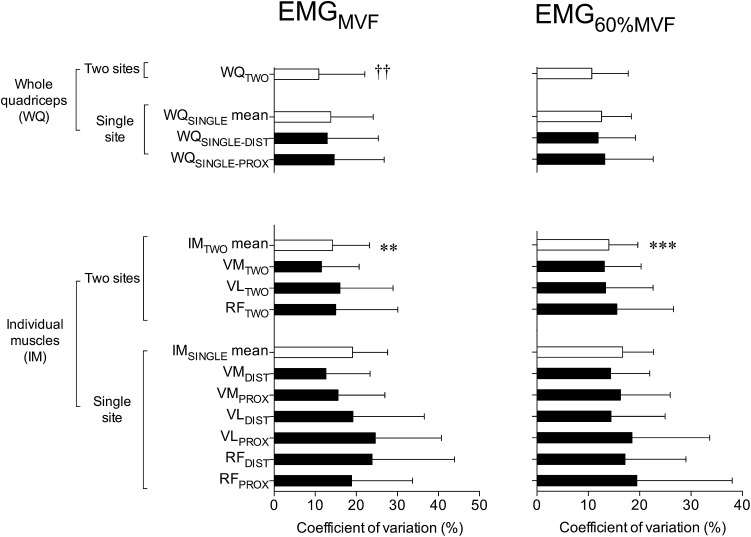
Within-participant coefficient of variation (CV_W_) for root mean square surface electromyography (sEMG) during a 500 ms time period surrounding maximum voluntary force (EMG_MVF_, *left*) and during a submaximum contraction at 60% of maximum force production (EMG_60%MVF_, *right*). Data are shown for single sEMG recording sites and the mean of two sites for individual muscles (IM), as well as whole quadriceps (WQ). White bars indicate calculated mean values independent of location/site/muscle except WQ_TWO_ which incorporates measures from all six sEMG recording sites. *VM* vastus medialis, *VL* vastus lateralis, *RF* rectus femoris, _*DIST*_ distal sEMG recording site, _*PROX*_ proximal sEMG recording site. Differences in CV_W_ were determined from Wilcoxon signed-rank tests as follows: ***significantly lower than IM_SINGLE_ mean (*P* < 0.001); **significantly lower than IM_SINGLE_ mean (*P* < 0.01); ††significantly lower than WQ_SINGLE_ mean (*P* < 0.01). Data are mean ± SD

**Fig. 3:**
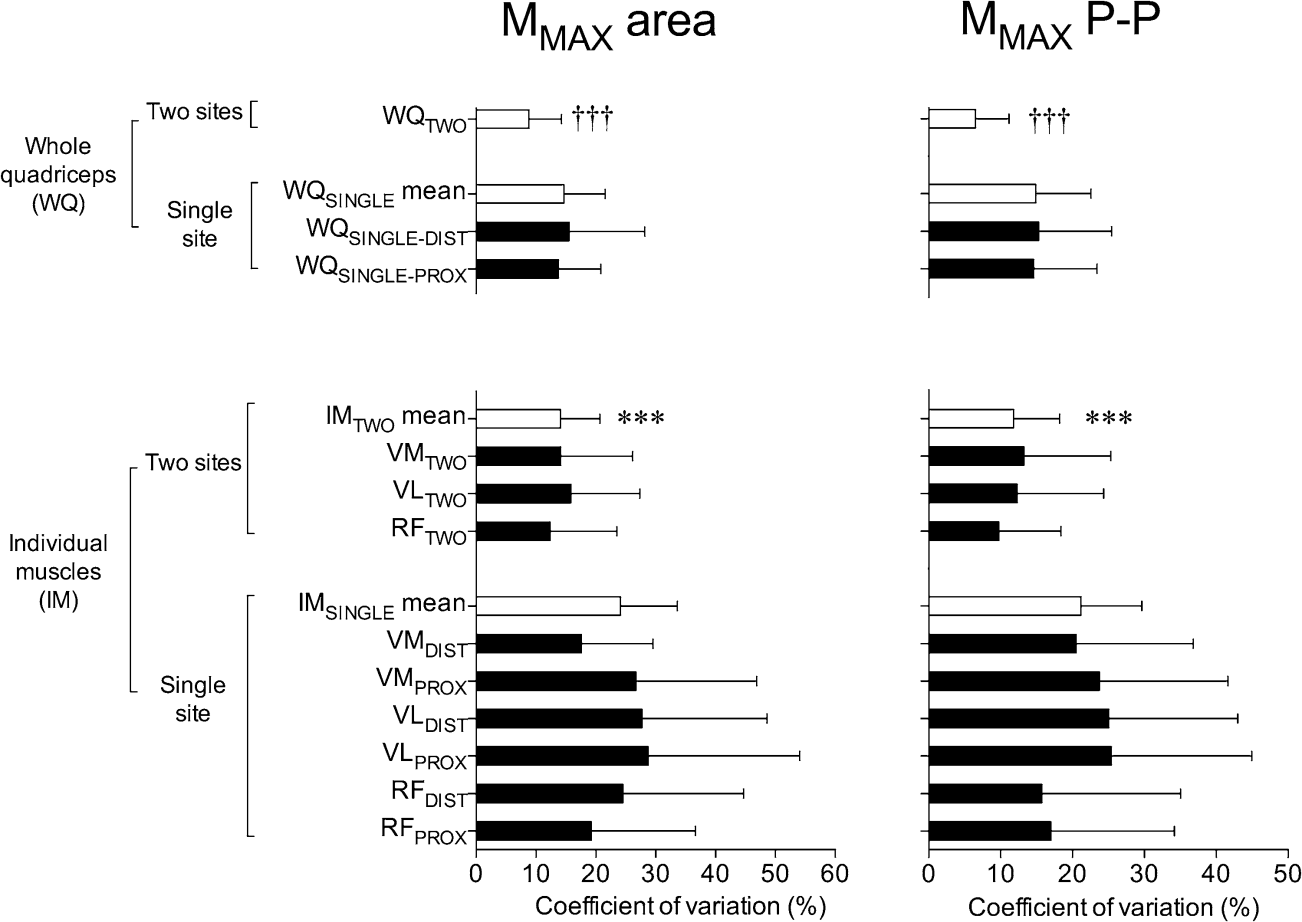
Within-participant coefficient of variation (CV_W_) for surface electromyography (sEMG) parameters [M_MAX_ area, *left*; and M_MAX_ peak-to-peak (P–P) amplitude, *right*] during evoked twitch contractions. Data are shown for single sEMG recording sites and the mean of two sites for individual muscles (IM), as well as whole quadriceps (WQ). White bars indicate calculated mean values independent of location/site/muscle except WQ_TWO_ which incorporates measures from all six sEMG recording sites. *VM* vastus medialis, *VL* vastus lateralis, *RF* rectus femoris, _*DIST*_ distal sEMG recording site, _*PROX*_ proximal sEMG recording site. Differences in CV_W_ were determined from Wilcoxon signed-rank tests as follows: ***significantly lower than IM_SINGLE_ mean (*P* < 0.001); †††significantly lower than WQ_SINGLE_ mean (*P* < 0.001). Data are mean ± SD

EMG data from session 1 and 2 are displayed in Table 1. When averaged across the individual quadriceps muscles, the CV_W_ of two site EMG measurements (IM_TWO_ mean) was significantly lower than for single site measurements (IM_SINGLE_ mean) for voluntary (EMG_MVF_: Wilcoxon *P* = 0.002; and EMG_60%MVF_ *P* < 0.001; Fig. 2) and evoked (M_MAX_ area: *P* < 0.001; and M_MAX_ P–P: *P* < 0.001; Fig. 3) contractions. Representing 16–26% and 41–44% reductions in CV_W_ values for voluntary and evoked contractions, respectively. Mean ICC values were higher for two vs. single site EMG measurements (e.g. IM_TWO_ mean vs. IM_SINGLE_ mean) during both voluntary and evoked contractions (Table 2). Representing a 2–9% and 10–15% improvement in ICC values for voluntary and evoked contractions, respectively. For each of the individual muscles CV_W_ values were lower for two vs. single site measures (e.g. VM_TWO_ vs. VM_SINGLE_ mean, VL_TWO_ vs. VL_SINGLE_ mean, RF_TWO_ vs. RF_SINGLE_ mean) during evoked contractions (M_MAX_ area and M_MAX_ P–P: Wilcoxon 0.001 < *P* ≤ 0.009). CV_W_ values were also lower or tended to be lower for VM_TWO_ vs. VM_SINGLE_ mean (EMG_MVF_ and EMG_60%MVF_: Wilcoxon 0.002 ≤ *P* ≤ 0.013) and VL_TWO_ vs. VL_SINGLE_ mean (EMG_MVF_: *P* = 0.062; and EMG_60%MVF_: *P* = 0.031) during voluntary contractions. CV_W_ values were 15–30% lower for RF_TWO_ vs. RF_SINGLE_ mean during voluntary contractions but this did not reach significance (EMG_MVF_: Wilcoxon *P* = 0.218; and EMG_60%MVF_: *P* = 0.100).

**Table 1:** Voluntary maximum (EMG_MVF_), voluntary submaximum (EMG_60%MVF_) and evoked [M_MAX_ area and M_MAX_ peak-to-peak (P–P)] surface EMG parameters measured on two separate test days

		EMG_MVF_ (mV)		EMG_60%MVF_ (mV)		M_MAX_ area (mV.s^− 1^)		M_MAX_ P-P (mV)	
	Test day	1	2	1	2	1	2	1	2
Whole quadriceps									
Two sites	WQ_TWO_	0.17 ± 0.06	0.18 ± 0.06	0.09 ± 0.03	0.08 ± 0.03	0.009 ± 0.004	0.009 ± 0.004	2.34 ± 1.04	2.32 ± 0.99
Single site	WQ_SINGLE−DIST_	0.18 ± 0.05	0.19 ± 0.07	0.09 ± 0.03	0.08 ± 0.03	0.011 ± 0.004	0.010 ± 0.004	2.66 ± 1.27	2.56 ± 0.91
	WQ_SINGLE−PROX_	0.16 ± 0.08	0.16 ± 0.07	0.08 ± 0.04	0.08 ± 0.03	0.008 ± 0.003	0.008 ± 0.004	2.01 ± 1.02	2.07 ± 1.19
Individual muscles									
Two sites	VM_TWO_	0.20 ± 0.07	0.19 ± 0.08	0.09 ± 0.03	0.09 ± 0.03	0.013 ± 0.005	0.012 ± 0.006	3.13 ± 1.66	2.85 ± 1.34
	VL_TWO_	0.19 ± 0.09	0.20 ± 0.08	0.10 ± 0.05	0.10 ± 0.04	0.010 ± 0.005	0.010 ± 0.005	2.63 ± 1.51	2.87 ± 1.56
	RF_TWO_	0.12 ± 0.05	0.13 ± 0.07	0.07 ± 0.03	0.06 ± 0.03	0.005 ± 0.003	0.005 ± 0.002	1.25 ± 0.57	1.22 ± 0.62
Single site	VM_DIST_	0.24 ± 0.09	0.24 ± 0.10	0.11 ± 0.04	0.11 ± 0.05	0.017 ± 0.007	0.016 ± 0.007	4.31 ± 2.25	3.79 ± 1.59
	VM_PROX_	0.15 ± 0.08	0.14 ± 0.08	0.07 ± 0.04	0.06 ± 0.03	0.009 ± 0.005	0.008 ± 0.006	1.95 ± 1.30	1.91 ± 1.74
	VL_DIST_	0.18 ± 0.07	0.19 ± 0.09	0.09 ± 0.04	0.08 ± 0.04	0.010 ± 0.006	0.009 ± 0.005	2.45 ± 1.73	2.64 ± 1.74
	VL_PROX_	0.19 ± 0.13	0.21 ± 0.09	0.10 ± 0.07	0.11 ± 0.05	0.010 ± 0.007	0.011 ± 0.006	2.81 ± 2.01	3.11 ± 1.61
	RF_DIST_	0.13 ± 0.06	0.14 ± 0.09	0.07 ± 0.04	0.06 ± 0.03	0.005 ± 0.002	0.005 ± 0.003	1.23 ± 0.53	1.24 ± 0.67
	RF_PROX_	0.12 ± 0.05	0.13 ± 0.07	0.07 ± 0.04	0.06 ± 0.03	0.005 ± 0.003	0.005 ± 0.003	1.27 ± 0.78	1.20 ± 0.74

Data are mean ± SD (*n* = 20)

*VM* vastus medialis, *VL* vastus lateralis, *RF* rectus femoris, _*DIST*_ distal sEMG recording site, _*PROX*_ proximal sEMG recording site

**Table 2:** Between-session intraclass correlation coefficient (ICC) and classifications for single (_SINGLE_) and average of two sites (_TWO_) sEMG measurements from individual muscles (IM) and the whole quadriceps (WQ) during maximum (EMG_MVF_) and submaximum (EMG_60%MVF_) voluntary contractions (500 ms epochs), as well as during evoked twitch contractions [M_MAX_ area and M_MAX_ peak-to-peak (P–P amplitude)]

	EMG_MVF_	EMG_60%MVF_	M_MAX_ area	M_MAX_ P-P
Whole quadriceps				
Two sites				
WQ_TWO_	0.851	0.943	0.960	0.984
	“High”	“Very high”	“Very high”	“Very high”
Single site				
WQ_SINGLE_ mean	0.797	0.927	0.885	0.919
	“High”	“Very high”	“High”	“Very high”
WQ_SINGLE−DIST_	0.750	0.929	0.836	0.887
	“High”	“Very high”	“High”	“High”
WQ_SINGLE−PROX_	0.843	0.924	0.934	0.951
	“High”	“Very high”	“Very high”	“Very high”
Individual muscles				
Two sites				
IM_TWO_ mean	0.875	0.925	0.923	0.953
	“High”	“Very high”	“Very high”	“Very high”
VM_TWO_	0.893	0.893	0.910	0.935
	“High”	“High”	“Very high”	“Very high”
VL_TWO_	0.879	0.937	0.916	0.963
	“High”	“Very high”	“Very high”	“Very high”
RF_TWO_	0.853	0.946	0.944	0.962
	“High”	“Very high”	“Very high”	“Very high”
Single site				
IM_SINGLE_ mean	0.801	0.904	0.806	0.870
	“High”	“Very high”	“High”	“High”
VM_DIST_	0.868	0.870	0.865	0.814
	“High”	“High”	“High”	“High”
VM_PROX_	0.923	0.920	0.822	0.91
	“Very high”	“Very high”	“High”	“Very high”
VL_DIST_	0.637	0.931	0.684	0.828
	“Moderate”	“Very high”	“Moderate”	“High”
VL_PROX_	0.832	0.895	0.802	0.887
	“High”	“High”	“High”	“High”
RF_DIST_	0.665	0.915	0.769	0.882
	“Moderate”	“Very high”	“High”	“High”
RF_PROX_	0.880	0.895	0.896	0.900
	“High”	“High”	“High”	“Very high”

*VM* vastus medialis, *VL* vastus lateralis, *RF* rectus femoris, _*DIST*_ distal sEMG recording site, _PROX_ proximal *sEMG* recording site

Whole quadriceps measurements from two sites displayed lower CV_W_ values than for single sites (e.g. WQ_TWO_ vs. WQ_SINGLE_ mean) for maximum voluntary (EMG_MVF_: Wilcoxon *P* = 0.002; Fig. 2) and evoked (M_MAX_ area: *P* < 0.001; and M_MAX_ P–P: *P* < 0.001; Fig. 3) contractions. Thereby, representing 21% and 40–56% reductions in CV_W_ values for maximum voluntary and evoked contractions, respectively. CV_W_ values were 15% lower for WQ_TWO_ vs. WQ_SINGLE_ mean during submaximum contractions but this did not reach statistical significance (EMG_60%MVF_: Wilcoxon *P* = 0.121). Whole quadriceps ICC values were greater for two site vs. single site EMG measurements (e.g. WQ_TWO_ vs. WQ_SINGLE_ mean) during voluntary and evoked contractions (Table 2). Representing 2–7% and 7–8% improvements in ICC values for voluntary and evoked contractions, respectively.

Additionally, the CV_W_ for the whole quadriceps were lower than those of the individual muscle for both single and two site measures (e.g. WQ_SINGLE_ mean vs. IM_SINGLE_ mean and WQ_TWO_ vs. IM_TWO_ mean) for voluntary (EMG_MVF_: Wilcoxon 0.001 < *P* ≤ 0.007; and EMG_60%MVF_: 0.001 < *P* ≤ 0.006; Fig. 2) and evoked (M_MAX_ area: *P* ≤ 0.001; and M_MAX_ P–P: [both] P = 0.002; Fig. 2) contractions. ICC values were 3–10% greater for whole quadriceps vs. single quadriceps EMG measurements for single (WQ_SINGLE_ mean vs. IM_SINGLE_ mean) and two (WQ_TWO_ vs. IM_TWO_ mean) site measures for evoked contraction parameters (M_MAX_ area and M_MAX_ P-P). During voluntary contractions ICC values were similar (1–3% difference) for single (WQ_SINGLE_ mean vs. IM_SINGLE_ mean) and two (WQ_TWO_ vs. IM_TWO_ mean) site measures (Table 2).

## Discussion

This study compared the between-session reliability (both within-participant absolute reliability and between-participant relative reliability) of sEMG amplitude measurements derived from single vs. average of two recording sites during maximum (EMG_MVF_) and submaximum (EMG_60%MVF_) voluntary, as well as electrically evoked (M_MAX_ area and M_MAX_ P–P) contractions for individual muscles and the whole quadriceps. The use of two vs. single recording sites improved within-participant absolute reliability (15–56% reduction in CV_W_) for individual muscles and whole quadriceps measurements during voluntary and evoked contractions. The results of this study indicate that quantifying voluntary and evoked sEMG measures from two, rather than single, recording sites substantially improved CV_W_ values for these variables. Therefore, it is strongly recommended that quantitative clinical and research measurements of sEMG amplitude, particularly those focusing on within-participant changes, record and average across two sites when addressing large locomotory muscles.

It appears that the enhanced within-participant absolute reliability of the sEMG parameters derived from averaging across two recording sites vs. single site measures in the present investigation was likely due to: quantifying electrical activity from a greater proportion of the motor unit pool; and the statistical effect of increasing the number of measurements performed. Measuring a greater proportion of the motor unit pool might be expected to provide a better, and more stable reflection of the whole muscle or overall muscle group. Averaging across two sites, may also exert a statistical effect simply by reducing measurement variability compared to recording from only one location on the muscle. Single site EMG reliability values of the present investigation were comparable to those reported from several previous studies conducting voluntary (Yang and Winter 1983; Mathur et al. 2005; Ball and Scurr 2010; Fauth et al. 2010; Buckthorpe et al. 2012; Rota et al. 2013) and evoked (Gondin et al. 2005; Place et al. 2007; Buckthorpe et al. 2012) isometric contractions. The current study performed measures during isometric contractions but these findings of enhanced within-participant absolute reliability from two site recordings would be expected to translate to all types of contractions although this needs to be confirmed by future research.

A further consideration when recording sEMG from large individual muscles (such as the VM, VL, and RF) is that often measurements from only one or two muscles of an overall group are collected (Higbie et al. 1996; Häkkinen et al. 1998; Brandon et al. 2014; Trulsson et al. 2015). Individual muscle measures have sometimes been assumed to provide reliable representation of whole muscle group activation. However, the greater within-participant reliability of whole quadriceps vs. individual muscle measurements (for voluntary and evoked contractions, as well as single and two site measures) in the current study suggests calculating whole quadriceps sEMG measures is preferable to enhance absolute reliability vs. measuring one or two of the individual constituent muscles. Therefore, it is strongly suggested that when overall acute or chronic changes in quadriceps sEMG parameters are of interest that measures averaged across the VM, VL, and RF are used.

The placement of two EMG sensors on the same muscle may introduce cross-talk between the sensors, i.e. some commonality to the recorded signals. To reduce this possibility, we took the following approaches: used double differential EMG sensors that are known to have a smaller detection volume than single differential sensors (Stepp 2012); used sensors with small inter-electrode distance (10 mm) that are thought to minimize cross-talk (De Luca et al. 2012); performed measurements in the current study on some of the largest muscles in the human body of healthy young men; and spatially separated the two sensors in both proximo-distal and medio-lateral directions. Qualitatively, the signals from two sensors on the same muscle appeared to be independent, nevertheless it is possible that there could have been some cross-talk between sensors, but currently there is no accepted analytical procedure to assess the extent of cross-talk within an EMG signal (Farina et al. 2014). The observation that averaging the two signals improves the reliability of EMG amplitude measurements may indicate that the signals were substantially independent, although it is unknown if this finding of improved reliability was specific to the conditions (cohort, muscles and electrodes) of our study.

In conclusion, the use of two vs. single sEMG recording sites improved the within-participant reliability of sEMG parameters across a range of different contraction types (voluntary maximum, submaximum, and electrically evoked). The effects of using two recording sites to quantify sEMG measures had the greatest benefit for within-participant reliability (CV_W_), but also produced some small but consistent improvement in relative measures of reliability (ICC). This greater reliability would be expected to increase the sensitivity of sEMG measurements to detect changes within, and differences between individuals. In addition, whole quadriceps sEMG within-participant reliability was greater than that of the individual muscles for both single and two site measures. Given the importance of reliability for clinical and research applications of sEMG, it is recommended that when measuring large muscles, such as the quadriceps femoris, that sEMG parameters are quantified by taking mean measures across two distinct recording sites before reporting absolute EMG values or normalising data.

## Abbreviations

_60%MVF_: Sixty percent of maximum voluntary force
CV_W_: Within-participant coefficient of variability
_DIST_: Distal recording site
sEMG: Surface electromyography
ICC: Intraclass correlation coefficient
IM: Individual muscle
M_MAX_: Supra-maximal M-wave
MVF: Maximum voluntary force
_PROX_: Proximal recording site
RF: Rectus femoris
VL: Vastus lateralis
VM: Vastus medialis
WQ: Whole quadriceps
